# Efficacy and Safety of Preoperative vs. Intraoperative Computed Tomography-Guided Lung Tumor Localization: A Randomized Controlled Trial

**DOI:** 10.3389/fsurg.2021.809908

**Published:** 2022-01-07

**Authors:** Hsin-Yueh Fang, Kuei-An Chen, Yu-Wen Wen, Chih-Tsung Wen, Kuang-Tse Pan, Chien-Hung Chiu, Ming-Ju Hsieh, Yin-Kai Chao

**Affiliations:** ^1^Division of Thoracic Surgery, Chang Gung Memorial Hospital-Linkou, College of Medicine, Chang Gung University, Taoyuan, Taiwan; ^2^Department of Medical Imaging and Intervention, Chang Gung Memorial Hospital-Linkou, College of Medicine, Chang Gung University, Taoyuan, Taiwan; ^3^Clinical Informatics and Medical Statistics Research Center, Chang Gung University, Taoyuan, Taiwan

**Keywords:** solitary pulmonary nodules, localization, ARTIS zeego, hybrid operating room, lung tumor

## Abstract

**Background:** Thoracoscopic removal of small pulmonary nodules is traditionally accomplished through a two-step approach—with lesion localization in a CT suite as the first step followed by lesion removal in an operating room as the second step. While the advent of hybrid operating rooms (HORs) has fostered our ability to offer a more patient-tailored approach that allows simultaneous localization and removal of small pulmonary nodules within a single-step, randomized controlled trials (RCTs) that compared the two techniques (two- vs. single-step) are still lacking.

**Methods:** This is a RCT conducted in an academic hospital in Taiwan between October 2018 and December 2019. To compare the outcomes of traditional two-step preoperative CT-guided small pulmonary nodule localization followed by lesion removal vs. single-step intraoperative CT-guided lesion localization with simultaneous removal performed by a dedicated team of thoracic surgeons. The analysis was conducted in an intention-to-treat fashion. The primary study endpoint was the time required for lesion localization. Secondary endpoints included radiation doses, other procedural time indices, and complication rates.

**Results:** A total of 24 and 25 patients who received the single- and two-step approach, respectively, were included in the final analysis. The time required for lesion localization was significantly shorter for patients who underwent the single-step procedure (median: 13 min) compared with the two step-procedure (median: 32 min, *p* < 0.001). Similarly, the radiation dose was significantly lower for the former than the latter (median: 5.64 vs. 10.65 mSv, respectively, *p* = 0.001).

**Conclusions:** The single-step procedure performed in a hybrid operating room resulted in a simultaneous reduction of both localization procedural time and radiation exposure.

## Introduction

The use of low-dose computed tomography (CT) for lung cancer screening has become increasingly popular in recent years. As a result, the number of asymptomatic patients referred to thoracic surgeons because of suspected lung nodules in need of surgical excision has been growing steadily (1, 2). Unfortunately, these pulmonary lesions are frequently thoracoscopically invisible and impalpable. In this scenario, a two-step approach—with percutaneous preoperative lesion localization in a CT suite as the first step followed by lesion removal in an operating room as the second step is commonly utilized to avoid unplanned conversion to open surgery during video-assisted thoracoscopic surgery (VATS) (3, 4).

However, this two-step workflow is operationally limited by time constraints and strict coordination required between two distinct teams (i.e., radiologists and surgeons who are separately in charge of lesion localization and removal, respectively) (5, 6). A prolonged time interval between localization and excision (termed “time at risk”) remains a substantial shortcoming associated with patient distress and an increased risk of marker failure (e.g., wire dislodgement or dye fading) (5, 6).

The advent of hybrid operating rooms (HORs) has fostered our ability to offer a more patient-tailored approach that allows simultaneous localization and removal of small pulmonary nodules within a single-step produce entirely performed by thoracic surgeons (7). This technique—which relies on intraoperative computed tomography (IOCT)-guided simultaneous lesion localization and removal—has been pioneered by our center as of 2015 (7). We have previously shown that the single-step approach is characterized by a significant learning curve as demonstrated by decreased localization time and radiation exposure occurring with increased surgical experience (8, 9). However, these data had a pilot nature and randomized controlled trials (RCTs) that compared the two localization techniques (two- vs. single-step) are still lacking. This RCT was therefore undertaken to analyze the outcomes of traditional two-step preoperative computed tomography (POCT)-guided small pulmonary nodule localization followed by lesion removal vs. single-step IOCT-guided simultaneous lesion localization and removal performed by a dedicated team of thoracic surgeons.

## Materials and Methods

### Study Design

This single-center, open-label, investigator-initiated, investigator-driven RCT was conducted in accordance with the Declaration of Helsinki and the provisions set forth in the GCP guidelines. The study protocol—which has been previously described in detail (*ClinicalTrials.gov* identifier: NCT03395964) (10)—was approved by the Ethics Committee of the Chang Gung Memorial Hospital (approval number: 201600671A3). All participants provided written informed consent.

### Participants

Patients referred for localization and removal of small pulmonary nodules were eligible for enrollment. Subpleural cavitary lesions and ground glass nodules were localized regardless of their size and/or depth. Solid nodules underwent localization when they were small-sized (diameter <10 mm) and/or deeply located (distance from the visceral pleura >10 mm).

### Randomization and Quality Control

During outpatient screening, patients who met the eligibility criteria were informed about the study and the different procedures for lesion localization and removal. At least 1 week was left for a decision to be made regarding inclusion in the trial. After obtaining written informed consent, patients were randomly assigned (1:1 ratio) by the trial coordinator (Eric Chung) to receive either the two- (POCT) or the single-step (IOCT) localization procedure using sealed opaque envelopes containing computer-generated random numbers. All single-stage procedures with IOCT localization were performed by a single team of thoracic surgeons (HY Fan and YK Chao; previous experience: >150 IOCT procedures). Localization by POCT during the two-step procedure was carried out by two experienced radiologists (KT Pan and KA Chen; previous experience: >300 POCT procedures). All of the outcome data were prospectively collected by the trial coordinator.

### Two-Step Procedure: POCT-Guided Localization

Patient positioning within the CT scanner (GE HiSpeed, Milwaukee, WI, USA) was performed to achieve the shortest possible direct path from the skin to the lung nodule. The lesion was localized on 2.5-mm-thick images. When possible, a direct and vertical needle trajectory was followed to reach the target lesion. A careful skin cleansing process was performed at the puncture site. Under local anesthesia, a small skin incision was created using a scalpel and a 10.7-cm long, 20-gauge cannula needle housing a 20-cm long double-thorn hook wire was gradually inserted (DuaLok®, Bard Peripheral Vascular, Inc., Tempe, AZ, USA) through the chest wall. The procedure was performed under sequential CT guidance. Whenever possible, lung lesions were pierced through the cannula needle. When the needle tip was properly positioned within the lesion or in its close proximity, the hook wire was advanced along the cannula. Superficial lesions were localized through the injection of patent blue V (PBV; concentration: 2.5%) dye (0.5 mL, Guerbet, Aulnay-sous-Bois, France) or diluted indocyanine green (ICG) through a 22-gauge spinal needle (length: 8.9 cm) as previously described (11). The correct positioning of the hook wire with respect to the lung nodule was confirmed with an immediate follow-up CT scans. Following lesion localization, patients were transferred to the surgical ward.

### Single-Step Procedure: IOCT-Guided Localization

IOCT-guided localization was performed in a HOR equipped with a C-arm cone-beam computed tomography (CBCT; ARTIS zeego; Siemens Healthcare GmbH, Erlangen, Germany) and a Magnus surgical table (Maquet Medical Systems, Wayne, NJ, USA). The procedural workflow has been previously described in detail (9). In brief, patients were placed in the lateral decubitus position after induction of general anesthesia. During end-inspiratory breath-holding, an initial scan for surgical planning was acquired using a 6-s acquisition protocol (6 s DynaCT Body). The entering trajectory was modeled in the isotropic data set under the syngo Needle Guidance of a syngo X-Workplace (Siemens Healthcare GmbH). The needle trajectory was initially laid out by marking the entry and target points; visualization of the needle entry point and angulation was accomplished by projecting a laser-target cross onto the patient's surface. Under three-dimensional laser-guidance and guided fluoroscopy, an 18-gauge marker needle was introduced into the patient's thorax during end-inspiratory breath-holding. Needle orientation and positioning were both corrected by projecting the planned, virtual needle trajectory onto the live fluoroscopic image. A fluoroscopic “bull eye” approach was used to introduce the needle and guide it onto the projected target. When the lesion was reached, the tumor was localized through the same approach by wire or dye as that in POCT-guided localization. The accuracy of tumor localization was confirmed by post-procedural CBCT scans.

### Surgical Treatment

Following VATS wedge resection (conducted either under hook wire or dye guidance), the excised lesion was subjected to frozen section examination. Lobectomy was performed in cases with a confirmed diagnosis of primary lung cancer. Patients with peripheral lung cancer of limited size (<2 cm) and adequate resection margins (either >2 cm or >tumor size) underwent sublobar resection.

### Outcome Assessment

**Figure 1:**
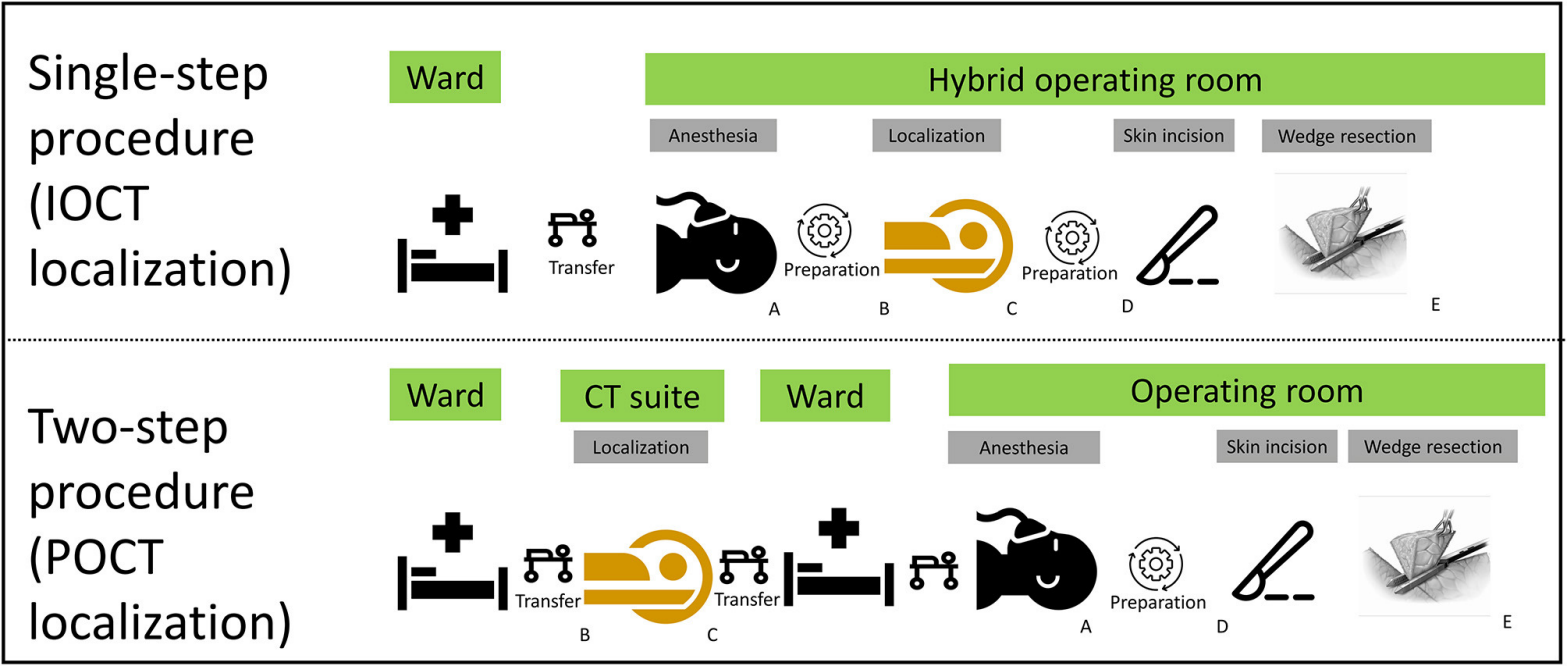
Procedural time stages. (A) Completion of anesthesia; (B) beginning of lesion localization; (C) end of lesion localization; (D) start of skin incision; (E) end of wedge resection.

The procedural stages of the single-step procedure (IOCT localization) were as follows: A, completion of anesthesia; B, beginning of lesion localization; C, end of lesion localization; D, start of skin incision; and E, end of wedge resection (Figure 1). The primary study endpoint was the time required for lesion localization (i.e., time elapsed from B to C). Secondary endpoints included (1) the rate of successful targeting during localization (defined as the number of successful targeting procedures divided by the number of all localization procedures), (2) the rate of successful localization in the operating field (defined as the number of successful targeting procedures minus the number of wire dislodgements or dye fading/spillage occurring in the operation field divided by the number of all localization procedures), (3) the duration of time at risk (i.e., time elapsed from C to D), (4) the time for surgical preparation (i.e., time elapsed from A to D), (5) the time from the start of skin incision to tumor resection (i.e., time elapsed from D to E), (6) the rate of conversion to thoracotomy, (7) the occurrence of complications (including pneumothorax and lung hemorrhage), and (8) the radiation dose. On analyzing procedural complications, large or small pneumothorax was defined according to the 2010 British Thoracic Society guidelines (i.e., distance between the lung margin and chest wall > 2 or ≤ 2 cm, respectively). The radiation dose was quantified by determining the effective dose (ED). The dose delivered by MDCT during the two-step procedure was determined using the dose length product (expressed as mGy/cm) and subsequently converted to the ED using a suitable conversion factor (0.014, mSvGy^−1^ cm^−1^). The dose delivered by both CBCT and fluoroscopy during the single-step procedure were determined using the dose area product and expressed as mGy/cm^2^. Two appropriate conversion factors (0.146 and 0.12 mSvGy^−1^ cm^−2^) was used to calculate the ED for CBCT and fluoroscopy, respectively (12, 13).

### Sample Size Calculation

The sample size was calculated based on the results of our previous retrospective study. The null hypothesis was that the time required for tumor localization would be equal for the single- vs. two-step approach (14). Based on a two-sample Student's *t*-test, a total sample size of 48 patients, 24 per arm, was required under the assumptions of an alpha error of 0.05, an 80% power, and a balanced trial design. Since a 10% total dropout rate was expected, this number was later expanded to permit enrollment of at least 27 patients per arm.

### Statistical Analysis

All analyses were performed according to the intention-to-treat (ITT) principle. All continuous variables are summarized as means (standard deviations) and median (first quartile–third quartile). Because of the skewed distribution of continuous variables, two-sample comparisons were carried out using the Wilcoxon rank-sum test. Categorical data are given as counts and percentages and analyzed with the Fisher's exact test. Statistical calculations were performed using R, version 4.1.0 (R Foundation for Statistical Computing, Vienna Austria), with all tests two-sided at a 5% level of significance.

## Results

### Characteristics of Patients and Pulmonary Nodules

**Figure 2:**
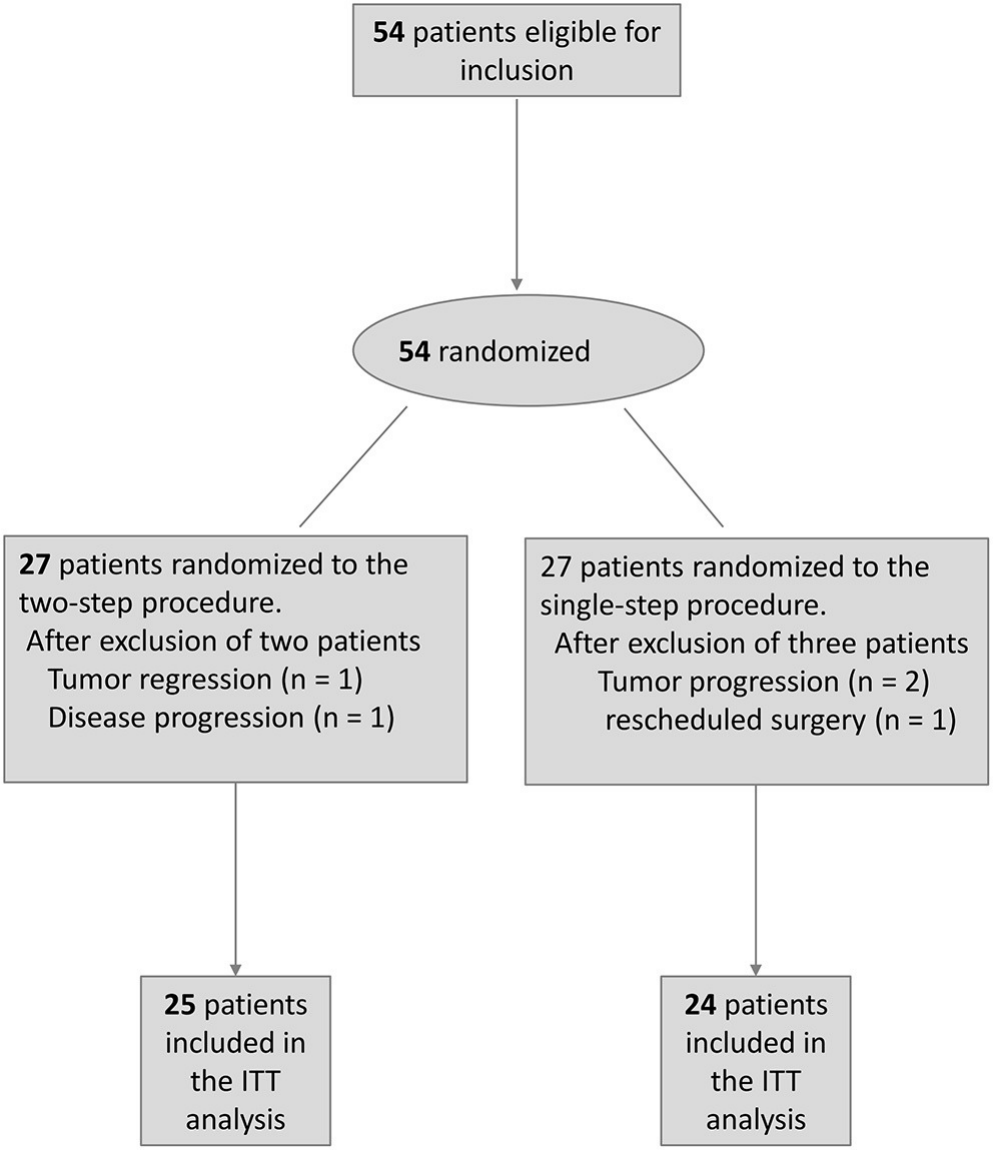
Flow of patients through the trial.

Between October 2018 and December 2019, 54 patients with small pulmonary nodules were randomized (1:1 ratio) to receive the two- or single-step procedure. Two patients randomized to the two-step procedure (POCT localization) and three to the single-step procedure (IOCT localization) did not ultimately undergo the planned lung tumor localization; therefore, they were excluded from the ITT analysis. Reasons for exclusion were as follows: tumor regression (*n* = 1), unexpected machine failure on the date of planned localization (*n* = 1), and tumor progression identified on pre-localization CT images that made futile the subsequent localization procedure (*n* = 3). Figure 2 depicts the flow of patients through the trial. A total of 49 patients were included in the final ITT analysis; of them, 25 and 24 participants were randomized to the two- and single-step localization procedure, respectively. There were no significant intergroup differences in terms of demographic and clinical characteristics (Table 1).

**Table 1:** General characteristics of patients in the two study arms.

	**Entire cohort**	**Two-step procedure (POCT localization)**	**Single-step procedure (IOCT localization)**	***P*-value**
Number of patients	49	25	24	
Age, years				
Mean (standard deviation)	56.53 (10.25)	55.72 (9.57)	57.38 (11.05)	
Median (Q1–Q3)	58 (50–64)	54 (49–61)	59.5 (50.75–65.25)	0.357
Sex, *n* (%)				0.387
Men	29 (59.2%)	13 (52%)	16 (66.7%)	
Women	20 (40.8%)	12 (48%)	8 (33.3%)	
ASA classification, *n* (%)				0.235
I–II	3 (6.1%)	3 (12%)	0 (0%)	
III	46 (93.9%)	22 (88%)	24 (100%)	
Time to treatment, days				
Mean (standard deviation)	23.12 (15.68)	23.04 (16.52)	23.21 (15.11)	
Median (Q1–Q3)	19 (12–26)	20 (12–26)	18 (12–31.5)	0.880
CT findings, *n* (%)				0.776
Solid nodule	28 (57.1%)	15 (60%)	13 (54.2%)	
Ground glass nodule	21 (42.9%)	10 (40%)	11 (45.8%)	
Lesion size on CT, mm				
Mean (standard deviation)	8.34 (3.56)	9 (3.94)	7.66 (3.06)	
Median (Q1–Q3)	7.2 (6–9)	8 (6.50–10.80)	7 (5.75–8.25)	0.265
Lesion location, *n* (%)				0.776
Right-sided	28 (57.1%)	15 (60%)	13 (54.2%)	
Left-sided	21 (42.9%)	10 (40%)	11 (45.8%)	
Distance to the pleural space, mm				
Mean (standard deviation)	8.45 (8.15)	8.64 (8.06)	8.25 (8.41)	
Median (Q1–Q3)	6.4 (1.0–13.3)	7.6 (3.00–13.3)	5 (1–13)	0.817
Depth-to-size ratio				
Mean (standard deviation)	1.18 (1.31)	1.24 (1.44)	1.11 (1.18)	
Median (Q1–Q3)	0.79 (0.28–1.71)	0.83 (0.31–1.85)	0.69 (0.16–1.46)	0.857

*POCT, preoperative computed tomography; IOCT, intraoperative computed tomography; Q1, first quartile; Q3, third quartile; ASA, American Society of Anesthesiologists*.

### Study Endpoints

Table 2 summarizes the results concerning the primary and secondary study endpoints. The time required for lesion localization (primary endpoint) was significantly shorter for patients who underwent the single-step procedure (median: 13 min) compared with the two step-procedure (median: 32 min, *p* < 0.001). Similarly, the time at risk between lesion localization and skin incision was significantly shorter for patients who received the single-step procedure (median: 13 min) compared with the two step-procedure (median: 245 min, *p* < 0.001). However, the time for surgical preparation was significantly longer for patients who received the single-step procedure (*p* < 0.001).

**Table 2:** Time-related outcomes in the two study arms.

**Parameter**	**Definition (see Figure 1)**	**Entire cohort**	**Two-step procedure (POCT localization) *n* = 25**	**Single-step procedure (IOCT localization) *n* = 24**	***P*-value**
Primary endpoint					
Localization time, min	B–C				
Mean (standard deviation)		23.29 (11.58)	31.84 (9.02)	14.38 (5.71)	
Median (Q1–Q3)		22 (13–33)	32 (25–38)	13 (11–16)	<0.001
Secondary endpoints					
Time at risk, min	C–D				
Mean (standard deviation)		130 (122.72)	241.40 (61.00)	13.96 (3.10)	
Median (Q1–Q3)		142 (13–245)	245 (193–266)	13 (12.00–15.25)	<0.001
Time from induction to incision, min	A–D				
Mean (standard deviation)		46.53 (22.12)	34.68 (19.86)	58.88 (17.26)	
Median (Q1–Q3)		44 (32–61)	32 (20–40)	55.5 (44.75–70.00)	<0.001
Time from incision to completion of wedge resection, min	D–E				
Mean (standard deviation)		16.55 (10.44)	16.36 (7.05)	16.75 (13.25)	
Median (Q1–Q3)		14 (11–19)	15 (11–22)	13.5 (11.75–16.5)	0.389
Time from anesthesia induction to completion of wedge resection, min	A–E				
Mean (standard deviation)		63.08 (23.99)	51.04 (21.29)	75.63 (20.16)	
Median (Q1–Q3)		59 (49–76)	51 (37–55)	76 (59–83.5)	<0.001

*POCT, preoperative computed tomography; IOCT, intraoperative computed tomography, Q1, first quartile; Q3, third quartile*.

Table 3 shows the results pertaining to safety and other non-temporal endpoints. The number of scans required for lesion localization was significantly lower for the single-step procedure (median: 2) compared with the two step-procedure (median: 12, *p* < 0.001). Similarly, the radiation dose was significantly lower for the former than the latter (median: 5.64 vs. 10.65 mSv, respectively, *p* = 0.001). On analyzing localization-related complications, no significant intergroup differences were observed with respect to the occurrence of pneumothorax and lung hemorrhage. All patients with procedural complications were symptomatic and were managed conservatively.

**Table 3:** Surgical variables in the two study arms.

	**Entire cohort**	**Two-step procedure (POCT localization) *n* = 25**	**Single-step procedure (IOCT localization) *n* = 24**	***P*-value**
Patient positioning for localization, *n* (%)				<0.001
Supine or prone	25 (51%)	25 (100%)	0 (0%)	
Lateral decubitus	24 (49%)	0 (0%)	24 (100%)	
Localization technique, *n* (%)				0.702
Hook wire	7 (14.3%)	3 (12%)	4 (16.7%)	
Dye	42 (85.7%)	22 (88%)	20 (83.3%)	
Post-procedural pneumothorax, *n* (%)	3 (6.1%)	3 (12%)	0 (0%)	0.235
Post-procedural lung hemorrhage, *n* (%)	1 (2%)	1 (4%)	0 (0%)	1.000
Effective dose, mSv	9.09 (6.21)	12.09 (7.17)	5.97 (2.65)	
Mean (standard deviation)	7.25 (4.45–10.82)	10.45 (6.64–16.38)	5.64 (3.98–8.32)	0.001
Median (Q1–Q3)				
Number of scans required for localization, *n* (%)				
Mean (standard deviation)	7.816 (6.28)	13.36 (3.66)	2.04 (0.20)	
Median (Q1–Q3)	9 (2–12)	12 (10–15)	2 (2)	<0.001
Surgical procedure for lesion excision, *n* (%)				0.490
Sublobar resection	47 (95.9%)	23 (92%)	24 (100%)	
Lobectomy	2 (4.1%)	2 (8%)	0 (0%)	
Successful targeting during localization, *n* (%)	49 (100%)	25 (100%)	24 (100%)	NA
Successful targeting during operation, *n* (%)	43 (87.8%)	20 (80%)	23 (95.8%)	0.189
Duration of post-operative hospital stay, days				
Mean (standard deviation)	2.69 (1.77)	2.92 (1.87)	2.46 (1.67)	
Median (Q1–Q3)	2 (2–3)	2 (2–3)	2 (1.75–3)	0.161
30-day readmissions after discharge, *n* (%)	1 (2%)	1 (4%)	0 (0%)	1.0

*POCT, preoperative computed tomography; IOCT, intraoperative computed tomography; Q1, first quartile; Q3, third quartile; NA, not available*.

Upon thoracoscopic exploration, marker failure was found to occur in five and two patients who received the two- and single-step procedure, respectively. In the former group, two cases of dye spillage and three of dye fading were observed. Both failures in the latter group were due to dye spillage. No patient who experienced marker failure required conversion to open thoracotomy. Thoracoscopic resection was carried out under needle puncture guidance. The median time from skin incision to the completion of wedge resection did not show significant intergroup differences. The postoperative course was uneventful. The median hospital stay after surgery was 2 days for both groups (*p* = 0.184). One patient experienced an unplanned readmission within the first 30 post-discharge days because of pleural effusion; the complication resolved spontaneously after conservative management.

## Discussion

This is, to our knowledge, the first RCT to compare two different procedural approaches for localization and resection of small, non-palpable lung tumors. We found that, using the single-step procedure in a HOR, thoracic surgeons were as able as radiologists to correctly localize pulmonary lesions. We also showed that, compared with the traditional two-step technique, the single-step approach reduced not only the time at risk but also the procedural time and radiation exposure. Collectively, our data indicate that thoracic surgeons may leverage the technical advantages offered by high-end HOR into a comprehensive diagnostic and therapeutic management of patients with small non-palpable lung nodules—without resorting to any separate radiological facility.

However, several aspects should be weighed by surgeons considering the implementation of the single-step approach proposed in our trial. First, the outcomes observed in the IOCT arm were likely the result of multiple operational refinements implemented over time. The IOCT technique was originally devised in 2016 with the goal of reproducing the methodology used by radiologists for POCT (7). Therefore, we initially attempted to place the patient in supine or prone position during localization followed by lateral decubitus repositioning for lesion removal (7). Unfortunately, this approach was time-consuming and clearly not applicable in a routine fashion within the HOR in the absence of further optimization. As of 2017, the procedural workflow was subjected to a refinement process aimed at eliminating certain passages with low added value (e.g., initial supine or prone positioning) (9). By using the lateral decubitus position only, we were able to accomplish both lesion localization and subsequent excision into an optimized single-step workflow. However, a significant shortcoming we faced when this approach was introduced was the risk of collision between the C-arm and the surgical table while we attempted to engage both the target lesion and the needle entry site within the same field of visualization (FOV). Under these circumstances, repeated patient repositioning and machine adjustments were required, which ultimately led to increased procedural time and radiation exposure. Currently, we have devised a detailed IOCT procedural manual that thoroughly illustrates how we conduct the entire IOCT procedure in the HOR. Notably, both the C-arm entry side and the needle trajectories are pre-planned to minimize the risk of collision. We also developed a detailed standard operating procedure checklist to facilitate standardization and replication of the proposed approach (Supplementary Figure 1). Some technical adjustments—including the reduction of surgical table thickness and modification of positioning according to the patient's anatomy—have been also proposed to further increase the proficiency of the single-step procedure and minimize the risk of repeated CBCT scanning (9, 15). These optimization steps may account for the markedly lower time required from completion of localization to skin incision in our study compared to other IOCT series.

Several caveats of our RCT need to be considered. First, the single-center nature of our trial poses a limitation regarding the ability to generalize our conclusions, and replication in independent samples is paramount for ensuring external validity. The implementation of the single-step procedure in our center dates back to 2016; however, key procedural details—including optimization of FOV design, HOR layout, and team coordination—were gradually refined over time, resulting in a significant learning curve. While our group was proactively involved in transferring the technical skills required for the single-step procedure to other surgical teams, none of them have yet completed the learning phase when the RCT was started. Second, it would have been interesting to compare the percutaneous CT-guided approach with other non-percutaneous methods (e.g., electromagnetic navigational bronchoscopy- or virtual bronchoscopy-guided marker injection); however, as mentioned above, per the protocol, only patients who had their lesion localized percutaneously were included. Finally, the reductions in both procedural time and time at risk observed with the single-step approach did not apparently translate into obvious clinical benefits—the only exception being a reduced radiation exposure; therefore, the final choice between the two options should still be guided by the most readily implementable strategy at each surgeon's facility.

## Conclusion

Using the single-step procedure in a HOR, thoracic surgeons were as able as radiologists to correctly localize pulmonary lesions. We also showed that, compared with the traditional two-step technique, the single-step approach reduced not only the time at risk but also the procedural time and radiation exposure.

## Data Availability Statement

The raw data supporting the conclusions of this article will be made available by the authors, without undue reservation.

## Ethics Statement

The studies involving human participants were reviewed and approved by Chang Gung Memorial Hospital. The patients/participants provided their written informed consent to participate in this study.

## Author Contributions

H-YF and Y-KC contributed to the conception and design of the study. H-YF and K-AC collected the study data, organized the dataset, and was in charge of follow-up. Y-WW was in charge of statistical analysis. H-YF, K-AC, and Y-KC prepared the first draft of the manuscript. All authors have made a significant contribution to the manuscript, read and approved the submitted version, and share responsibility for it.

## Funding

This study was financially supported by grants (CMRPG3F1813 and CMRPG3K0762) from the Chang Gung Memorial Hospital, Taiwan.

## Conflict of Interest

The authors declare that the research was conducted in the absence of any commercial or financial relationships that could be construed as a potential conflict of interest.

## Publisher's Note

All claims expressed in this article are solely those of the authors and do not necessarily represent those of their affiliated organizations, or those of the publisher, the editors and the reviewers. Any product that may be evaluated in this article, or claim that may be made by its manufacturer, is not guaranteed or endorsed by the publisher.

## Supplementary Material

Supplementary Figure 1 Standard operating procedure implemented for the single-step procedure (IOCT localization): operational checklist.
