# Supplementary figures and images for: Efficacy and Safety of Preoperative vs. Intraoperative Computed Tomography-Guided Lung Tumor Localization: A Randomized Controlled Trial
Source: Front Surg. 2022 Jan 7;8:809908. doi: 10.3389/fsurg.2021.809908 (PMC8782202; doi:10.3389/fsurg.2021.809908)

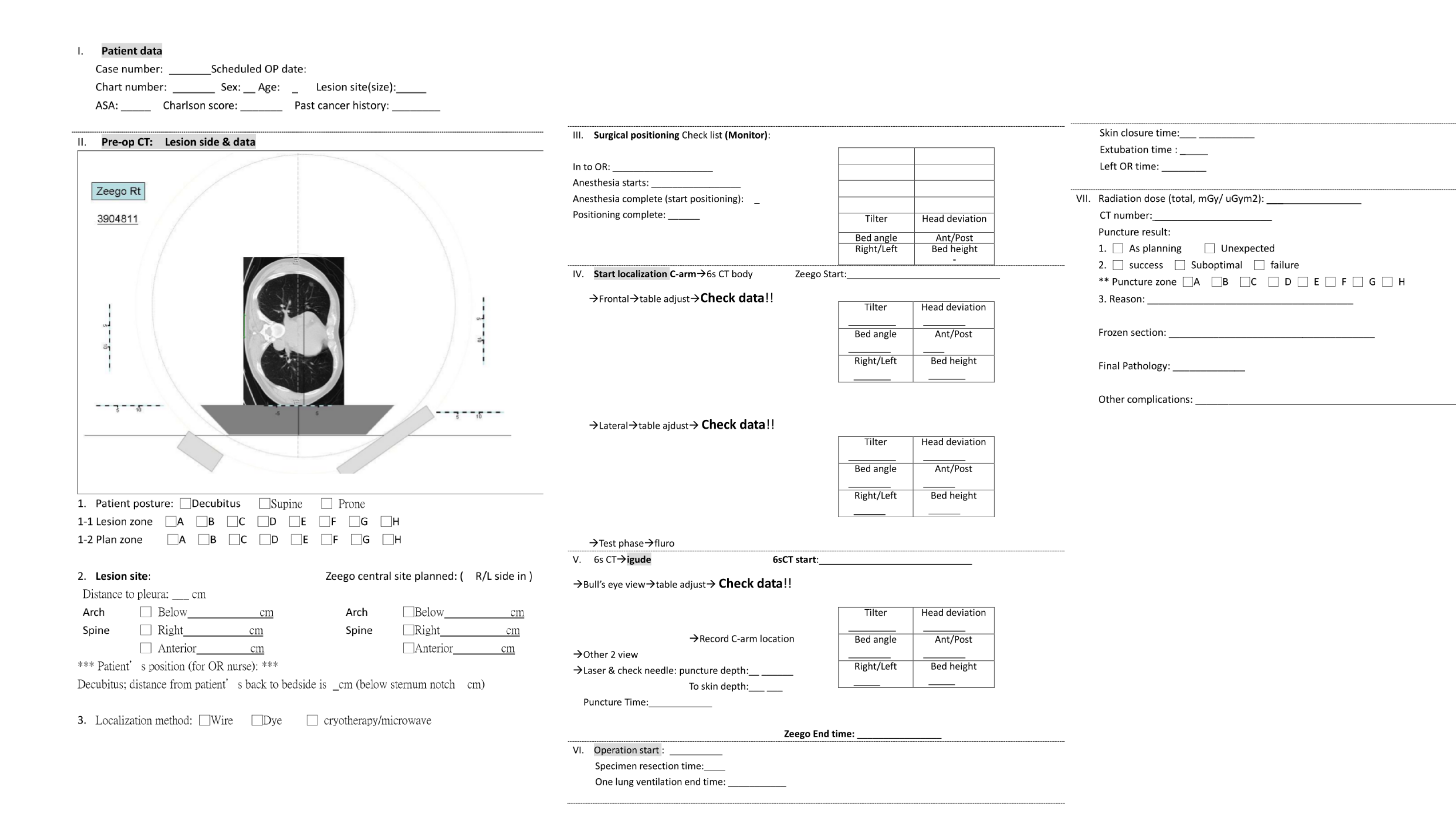

Supplement: Supplementary Figure 1 — Standard operating procedure implemented for the single-step procedure (IOCT localization): operational checklist. [file Image_1.TIF]
